# *miR-374b-5p* Modulates Melanoma Progression by Targeting VEGFC and Regulating MAPK Signaling in the Tumor Microenvironment

**DOI:** 10.3390/ijms27062854

**Published:** 2026-03-21

**Authors:** Zhen Chen, Fangjun Liu, Yixiao Cheng, Pengfei Li, Michael Rain Riggs, Wansheng Liu, Zhiwei Zhu

**Affiliations:** 1 College of Life Science, Shanxi Agricultural University, Taigu, Jinzhong 030801, China; 17861922495@163.com (Z.C.); 15735646830@163.com (F.L.); 18835678528@163.com (Y.C.); adamlpf@126.com (P.L.); 2 Department of Animal Science, Center for Reproductive Biology and Health (CRBH), College of Agricultural Sciences, The Pennsylvania State University, University Park, PA 16802, USA; riggs@psu.edu

**Keywords:** *miR-374b-5p*, VEGFC, MAPK, melanoma, tumor microenvironment

## Abstract

Melanoma is an aggressive skin cancer with high metastatic potential and poor long-term survival, highlighting the need for new therapeutic targets. Although microRNAs are critical regulators of tumor progression, the function of *miR-374b-5p* in melanoma remains poorly understood. Here, we identify *miR-374b-5p* as a tumor suppressor in melanoma cells. We show that *miR-374b-5p* directly targets *vascular endothelial growth factor C* (*Vegfc*) and is associated with changes in mitogen-activated protein kinase (MAPK) signaling, accompanied by reduced levels of phosphorylated extracellular signal-regulated kinase (pERK) and tyrosinase (TYR). Consistent with these observations, *miR-374b-5p* overexpression suppresses melanoma cell proliferation, migration, and invasion in vitro. Conditioned media from *miR-374b-5p*-overexpressing melanoma cells is also associated with changes in macrophage-related inflammatory markers, suggesting that these alterations are consistent with a shift toward a more pro-inflammatory macrophage phenotype. In a mouse model, *miR-374b-5p* overexpression significantly reduced tumor growth and angiogenesis, and downregulated the lymphangiogenic factor VEGFC. Together, these findings identify *miR-374b-5p* as a novel regulator of melanoma progression that acts through VEGFC-associated MAPK signaling and tumor microenvironment reprogramming, identifying *miR-374b-5p* as a promising therapeutic candidate for melanoma.

## 1. Introduction

Melanoma is an aggressive malignancy of melanocytes, with incidence rising globally due to factors like increased ultraviolet radiation exposure [1]. Despite advances in immunotherapy and targeted therapy, the long-term survival rate remains low because melanoma cells have a strong propensity for invasion and metastasis, primarily through the lymphatics and hematogenous pathways [2,3]. Tumor cells disseminate to distant tissues, establish secondary lesions, and ultimately render the disease difficult to cure.

Vascular endothelial growth factor C (VEGFC) is a key molecular driver of melanoma progression and metastasis, acting as a major regulator of tumor angiogenesis and lymphangiogenesis [4]. As a ligand for VEGFR-3 and VEGFR-2, VEGFC not only promotes lymphatic and blood vessel formation to supply nutrients and oxygen for tumor proliferation [5], but also enhances melanoma cell migration and invasiveness, accelerating their spread via vascular and lymphatic pathways [6,7]. In melanoma, VEGFC activation of VEGFR-2 and VEGFR-3 triggers downstream signals, particularly the mitogen-activated protein kinase (MAPK) signaling pathway [8,9,10,11]. Whereas MAPK signaling is tightly regulated in normal cells, persistent MAPK activation in melanoma-often driven by genetic mutations-leads to uncontrolled proliferation [12]. High VEGFC expression correlates with aggressive disease and poor therapeutic response, whereas inhibition of VEGFC-mediated signaling can effectively limit metastasis [13,14]. Thus, VEGFC-associated signaling pathways, including MAPK signaling, may represent potential targets for therapeutic intervention [4]. Several microRNAs (miRNAs), including *miR-145* [15] and *miR-205* [16], have been shown to modulate VEGFC expression, affecting melanoma initiation and progression.

miRNAs are endogenous 20–24 nt non-coding RNAs that regulate gene expression post-transcriptionally by binding to the messenger RNA (mRNA), controlling protein synthesis and cellular functions [17,18,19]. High-throughput sequencing transcriptome data have revealed distinct miRNA expression profiles between tumor and normal tissues, often associated with tumor stage and prognosis [20,21]. Among these, *miR-374b-5p* has emerged as a tumor suppressor across multiple cancers [22,23]. It inhibits cell proliferation and induces apoptosis in T-cell acute lymphoblastic leukemia by targeting *Akt1* and *Wnt-16* [24], and suppresses pancreatic cancer cell proliferation, migration, and invasion [25]. In non-small cell lung cancer, it exerts antitumor effects by targeting *FOXP1* and serves as a prognostic biomarker [26]. Tumor-derived miRNAs can also influence the tumor microenvironment by affecting immune cell behavior. For example, exosomal *miR-203*, released by tumors, promotes M1 macrophage polarization in prostate cancer [27], whereas *miR-4488*, from pancreatic neuroendocrine tumors, drives M2 macrophage polarization via RTN3/FABP5, facilitating liver metastasis [28]. However, the precise mechanisms by which *miR-374b-5p* exerts its antitumor effects in melanoma remain to be fully elucidated. Emerging evidence suggests that miRNAs can simultaneously modulate tumor cell-intrinsic signaling and reshape the tumor microenvironment; yet whether *miR-374b-5p* participates in multi-layered regulatory processes involving VEGFC-associated signaling, angiogenesis, and macrophage-related responses has yet to be systematically investigated. In this study, we verified the direct interaction between *miR-374b-5p* and *Vegfc* by bioinformatic prediction and dual-luciferase reporter assays. We found that *miR-374b-5p* targets *Vegfc* and is associated with modulation of MAPK signaling, suggesting a potential regulatory relationship between *miR-374b-5p* and VEGFC in melanoma progression. Using cellular functional assays (proliferation, migration, and invasion tests), we systematically investigated the impact of this axis on the biological behavior of melanoma cells, followed by in vivo validation using a murine melanoma model. We examined how *miR-374b-5p* may influence features of the tumor microenvironment, focusing on angiogenesis and macrophage polarization. Together, our in vitro and in vivo findings suggest that *miR-374b-5p* targets VEGFC and is associated with changes in MAPK signaling, melanoma progression, and features of the tumor microenvironment. These findings identify *miR-374b-5p* as a promising candidate for therapeutic intervention.

## 2. Results

### 2.1. Identification and Screening of Potential Target Genes of miR-374b-5p

**Figure 1:**
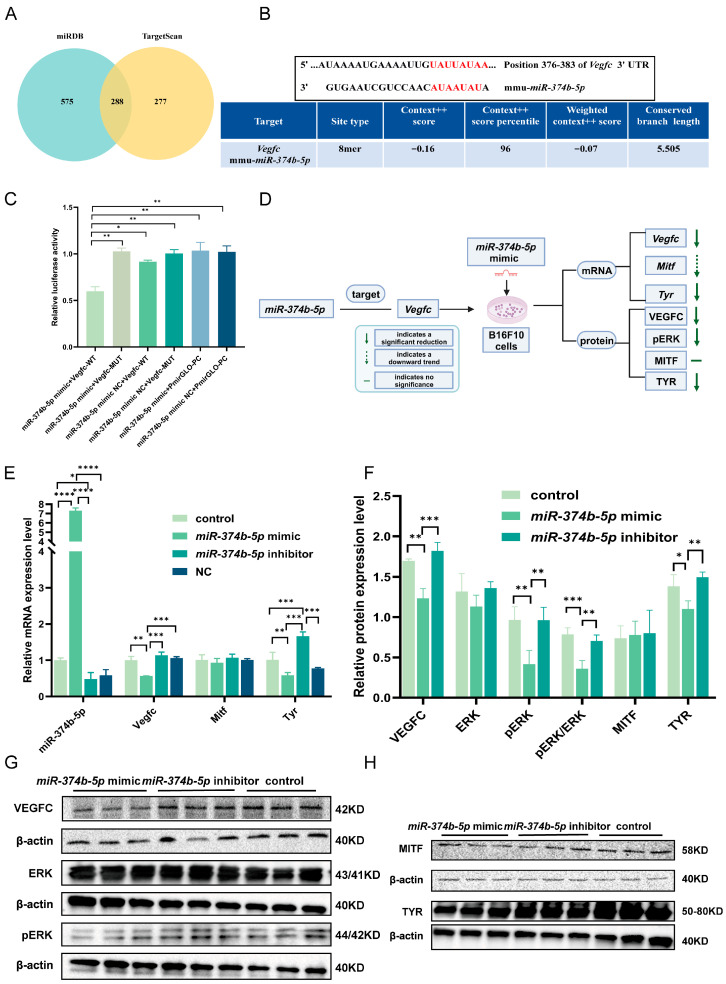
Validation of *miR-374b-5p* targeting *Vegfc* and its downstream pathway. (**A**) Intersection of *miR-374b-5p* target predictions: miRDB and TargetScan analysis. (**B**) Conserved target binding sites for *miR-374b-5p* and *Vegfc*. The red font indicates the targeted base pairs. (**C**) Dual luciferase assay for targeting between *miR-374b-5p* and *Vegfc* in 293T cells, with *Vegfc*-WT representing wild-type *Vegfc*, *Vegfc*-MUT representing *Vegfc* with mutated binding site and PmirGlo-PC serving as blank carrier, where * denotes *p* < 0.05, ** denotes *p* < 0.01 by one-way ANOVA Tukey’s multiple comparisons. (**D**) Schematic diagram of the targeted interaction between *miR-374b-5p* and *Vegfc* and its downstream regulatory mechanism. (**E**) Following transfection of *miR-374b-5p* into B16F10 cells, the relative expression levels of *miR-374b-5p*, *Vegfc*, *Mitf*, and *Tyr* were determined via qRT-PCR, where * denotes *p* < 0.05, ** denotes *p* < 0.01, *** denotes *p* < 0.001, and **** denotes *p* < 0.0001 by one-way ANOVA Tukey’s multiple comparisons. (**F**) Detection of the relative expression levels of VEGFC, ERK, pERK, pERK/ERK, MITF, and TYR proteins in B16F10 cells via Western blotting. (**G**) Representative Western blots showing VEGFC, ERK, pERK, and β-actin in B16F10 cells after transfection with *miR-374b-5p*. (**H**) Representative Western blots showing MITF, TYR, and β-actin in B16F10 cells after transfection with *miR-374b-5p*.

To identify *miR-374b-5p* target genes, two online databases, miRDB and TargetScan, were used. These databases predicted 863 and 565 potential target genes, respectively. The intersection yielded 288 common targets (Figure 1A), which were considered high-confidence candidates for further analysis. KEGG pathway enrichment of these 288 genes revealed significant enrichment in cancer-related pathways, including the MAPK, TGF-beta, and Ras signaling pathways (Supplementary Table S2). Among these, the MAPK signaling pathway attracted particular attention because of its well-established role in melanoma cell proliferation, survival, and metastasis [29,30]. Fourteen MAPK-related genes were predicted as targets of *miR-374b-5p* (Supplementary Table S2).

TargetScan 8.0 analysis of these 14 genes identified a predicted high-confidence 8-mer binding site for *mmu-miR-374b-5p* within the 3′UTR of *Vegfc* (Context++ score: −0.16; 96th percentile), ranking this site among the top 4% of all predicted *miR-374b-5p* targets (Figure 1B). Given its strong predicted binding score and its known involvement in melanoma-related processes such as angiogenesis and lymphangiogenesis, *Vegfc* was selected for further functional validation [31,32]. Cross-species sequence comparisons showed that the *miR-374b-5p-Vegfc* targeting interaction is highly conserved across humans, mice, and other vertebrates (Supplementary Figure S1B), suggesting evolutionary conservation and potential translational relevance.

Co-transfection of *miR-374b-5p* mimic + *Vegfc*-WT significantly altered luciferase activity compared with the mimic NC + *Vegfc*-WT control (Figure 1C, *p* < 0.05). The mimic + *Vegfc*-WT group also showed significantly different luciferase activity relative to the mimic + *Vegfc*-MUT group (Figure 1C, *p* < 0.01). Furthermore, luciferase activity in the mimic + *Vegfc*-WT group was significantly reduced compared with the mimic NC + *Vegfc*-MUT group (Figure 1C, *p* < 0.01). Collectively, these results support that *miR-374b-5p* directly interacts with the V*egfc* 3′UTR and represses reporter activity.

### 2.2. miR-374b-5p Influences VEGFC Expression and MAPK-Related Signaling in B16F10 Cells

To explore the potential role of *miR-374b-5p* in regulating VEGFC expression and MAPK-related signaling in melanoma cells in vitro (Figure 1D), we first assessed the transfection efficiency of *miR-374b-5p* in B16F10 cells using qRT-PCR. The results showed that *miR-374b-5p* expression was markedly higher in the *miR-374b-5p* mimic group compared with the control group (Figure 1E, *p* < 0.0001), confirming effective overexpression. In contrast, *miR-374b-5p* expression in the inhibitor group was significantly reduced relative to the control group (Figure 1E, *p <* 0.05). These results verify the successful transfection of *miR-374b-5p* mimic and inhibitor into B16F10 cells, and provide a solid foundation for subsequent functional studies.

We then detected the expression of the *miR-374b-5p* target gene, *Vegfc*, and two melanoma-related genes, *Mitf* and *Tyr*, using qRT-PCR. Compared to the control groups, the *miR-374b-5p* mimic group showed a significant reduction in *Vegfc* mRNA expression (Figure 1E, *p* < 0.01), whereas the inhibitor group exhibited a nonsignificant trend toward increased *Vegfc* expression (Figure 1E, *p* > 0.05). Although not statistically significant, *Mitf* mRNA levels displayed a decreasing trend in the mimic group relative to the other groups (Figure 1E, *p* > 0.05). In contrast, *Tyr* expression was significantly downregulated in the mimic group (Figure 1E, *p* < 0.01) and significantly upregulated in the inhibitor group compared with the control (Figure 1E, *p* < 0.001). These findings further support the inhibitory effect of *miR-374b-5p* on *Vegfc* expression and suggest an association with altered *Mitf* and *Tyr* expression.

Western blotting analysis further revealed that, compared with the control and inhibitor groups, the *miR-374b-5p* mimic significantly downregulated the protein expression of VEGFC (Figure 1F, G, *p* < 0.01, *p* < 0.001). Furthermore, we examined the protein expression levels of total ERK and found no significant differences among the groups (Figure 1F,G, *p* > 0.05), indicating that *miR-374b-5p* mimics do not affect the total protein level of ERK. However, compared with the control and inhibitor groups, the level of phosphorylated ERK (pERK) was significantly reduced in the *miR-374b-5p* mimic group (Figure 1F,G, *p* < 0.01, *p* < 0.01). In addition, the levels of pERK/ERK were significantly lower than those in the control group (Figure 1F,G, *p* < 0.001). These findings suggest that in B16F10 cells, overexpression of *miR-374b-5p* is associated with reduced VEGFC expression and decreased ERK phosphorylation, consistent with suppression of MAPK pathway activity. In contrast, MITF protein expression was not affected by *miR-374b-5p* (Figure 1F,H, *p* > 0.05). Compared with the inhibitor and control groups, TYR protein expression was significantly reduced in the mimic group (Figure 1F,H, *p* < 0.01, *p* < 0.05). Collectively, these results suggest that *miR-374b-5p* overexpression is associated with reduced VEGFC expression, decreased ERK phosphorylation, and lower TYR protein levels in B16F10 cells.

### 2.3. miR-374b-5p Impairs Malignant Phenotypes of B16F10 Cells to Inhibit Melanoma Progression

**Figure 2:**
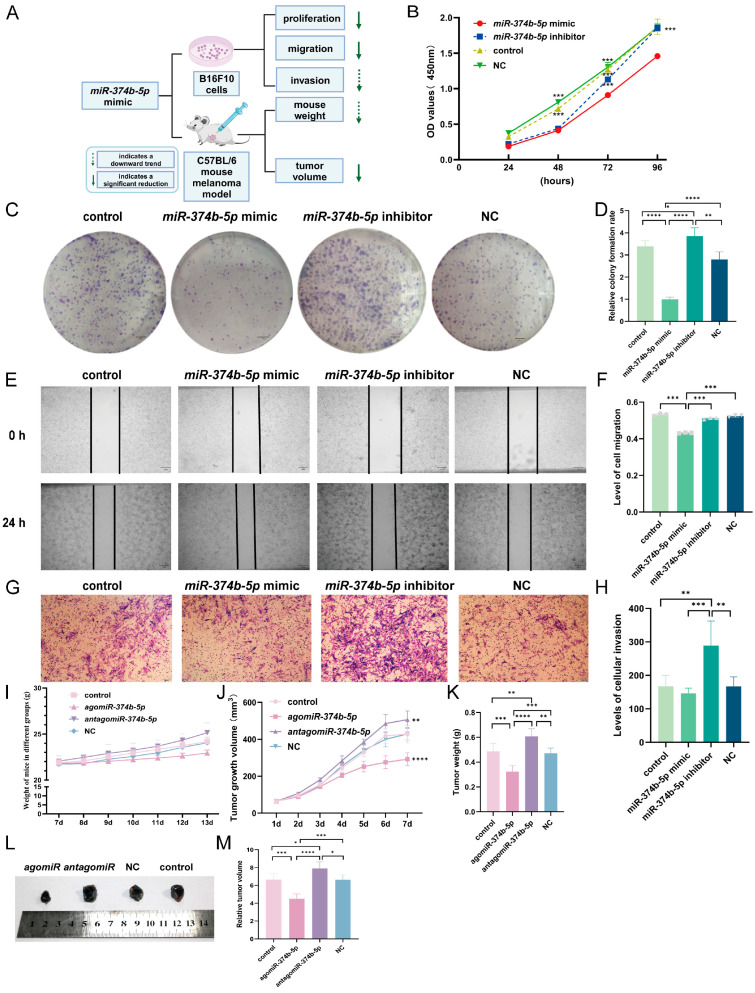
Impact of *miR-374b-5p* on B16F10 function and melanoma progression in vivo. (**A**) Schematic diagram of *miR-374b-5p* effects on B16F10 cell function and on body weight and tumor volume in a mouse melanoma model. (**B**) Effect of *miR-374b-5p* on the proliferation of B16F10 cells detected by CCK-8 assay. (**C**) Effect of *miR-374b-5p* on B16F10 cell proliferation was detected using the plate cloning assay. Scale bar: 100 μm. (**D**) Statistical plot of the plate cloning assay to detect the effect of *miR-374b-5p* on the proliferation of B16F10 cells. (**E**) Effect of *miR-374b-5p* on B16F10 cell migration as detected by scratch assay. Scale bar: 100 μm. (**F**) Statistical analysis of the effect of *miR-374b-5p* on B16F10 cell migration, as determined by the scratch assay. The gray symbols represent individual data values for each group. (**G**) Transwell assay was used to detect the effect of *miR-374b-5p* on B16F10 cell invasion. Scale bar: 100 μm. (**H**) Statistical plot of *miR-374b-5p* on incremental invasion of B16F10 cells detected by the Transwell method, where * denotes *p* < 0.05, ** denotes *p* < 0.01, *** denotes *p* < 0.001, and **** denotes *p* < 0.0001 by one-way ANOVA Tukey’s multiple comparisons. (**I**) Statistics of *miR-374b-5p* gene-related treatment on body weight in a mouse melanoma model. (**J**) Growth curves of subcutaneous melanoma in each group of mice. (**K**) Final tumor weights of subcutaneous melanoma in each group of mice. (**L**) Morphological images of subcutaneous melanoma in each group of mice, where “*agomiR*” represents “*agomiR-374b-5p*”, and “*antagomiR*” represents “*antagomiR-374b-5p*”. (**M**) Changes in relative tumor volume following *miR-374b-5p*-related therapy in a mouse melanoma model, where * denotes *p* < 0.05, ** denotes *p* < 0.01, *** denotes *p* < 0.001, and **** denotes *p* < 0.0001 by one-way ANOVA Tukey’s multiple comparisons.

To clarify the functional role of *miR-374b-5p* in B16F10 cells (Figure 2A), we first assessed cell proliferation using the CCK-8 assay by measuring the absorbance of cultured B16F10 cells at 24, 48, 72, and 96 h. The *miR-374b-5p* mimic group displayed significantly lower absorbance at 450 nm compared with control, inhibitor, and NC groups (Figure 2B, *p* < 0.001), indicating that the overexpression of *miR-374b-5p* strongly inhibited B16F10 cell proliferation.

Colony formation assays confirmed these results: mimic-transfected cells formed significantly fewer colonies than control, NC, and inhibitor groups (Figure 2C,D, *p* < 0.0001), whereas inhibitor-transfected cells produced more colonies than control (Figure 2C,D, *p* < 0.05).

We next assessed cell migration with a scratch assay. Photographs taken at 0 and 24 h showed that the *miR-374b-5p* mimic group exhibited significantly reduced migration compared with the control, inhibitor, and NC groups (Figure 2E,F, *p* < 0.001), and migration in the remaining groups was comparable (*p* > 0.05). This suggests that *miR-374b-5p* overexpression impairs cell motility.

Finally, we assessed the invasive ability of B16F10 cells. Cells in the inhibitor group showed significantly higher invasiveness than control (Figure 2G,H, *p* < 0.01), mimic (Figure 2G,H, *p* < 0.001), and NC (Figure 2G,H, *p* < 0.01) groups. Although the mimic group had slightly lower invasion than control, the difference was not statistically significant (Figure 2G,H, *p* > 0.05). This may reflect an existing inhibitory effect of endogenous *miR-374b-5p* on B16F10 cell invasion.

Collectively, these results indicate that *miR-374b-5p* suppresses the malignant phenotypes of B16F10 cells, including proliferation, migration, and invasion.

### 2.4. miR-374b-5p Suppresses Murine Melanoma Growth In Vivo

To clarify the in vivo role of *miR-374b-5p* in melanoma (Figure 2A), we established a murine tumor model by subcutaneously inoculating 1 × 10^6^ B16F10 cells into the axillary region of mice. *miR-374b-5p* treatments were initiated on day 7 after tumor implantation.

Throughout the treatment period, body weight was monitored daily to assess general health and treatment tolerance. Following administration of the corresponding *miR-374b-5p* reagents on day 7, mice in the *agomiR-374b-5p* group displayed reduced weight gain compared with the control group (Figure 2I, *p* > 0.05), consistent with effective suppression of tumor burden. In contrast, mice in the *antagomiR-374b-5p* group showed higher body weights than the control group (Figure 2I, *p* > 0.05), likely reflecting enhanced tumor growth when endogenous *miR-374b-5p* activity is inhibited.

During *miR-374b-5p* treatment, we measured the changes in subcutaneous melanoma volume in mice from each group on a daily basis. The results showed that tumor volume began to increase rapidly from day 3; by day 7, the tumor volume in the *agomiR-374b-5p* treatment group was already significantly smaller than that of the control group (Figure 2J, *p* < 0.0001), while the *antagomiR-374b-5p* group was significantly larger than the control group (Figure 2J, *p* < 0.01). At the experimental endpoint (day 14), after the mice were euthanized, we further measured the tumor weights in each group. We found that the average tumor weight in the *agomiR-374b-5p* group was significantly lower than in the control group (Figure 2K, *p* < 0.001), consistent with the trend of smaller tumor volumes; conversely, the *antagomiR-374b-5p* group showed the opposite trend.

On day 14, the relative tumor volume in the *agomiR-374b-5p* group was significantly smaller than that in the control group (Figure 2L,M, *p* < 0.001). In contrast, the *antagomiR-374b-5p* group exhibited a significant increase in tumor volume compared with the control group (Figure 2L,M, *p* < 0.05). These results demonstrate that *miR-374b-5p* acts as a suppressor of melanoma growth in vivo.

### 2.5. miR-374b-5p Downregulates VEGFC and MAPK-Related Effectors in Murine Melanoma Tumors

**Figure 3:**
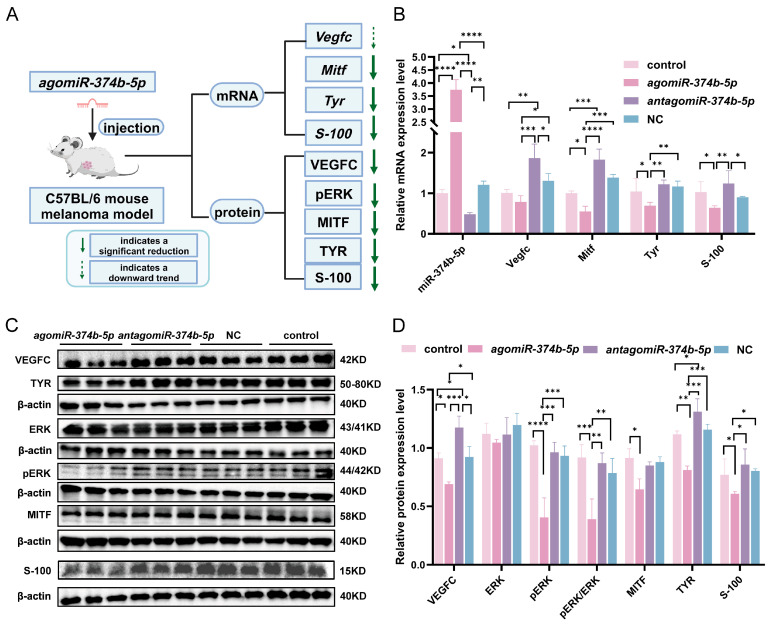
Effect of *miR-374b-5p* on *Vegfc* and its downstream proteins in a mouse melanoma model. (**A**) Schematic diagram of *miR-374b-5p*’s effects on *Vegfc* and its downstream proteins in a mouse melanoma model. (**B**) qRT-PCR analysis of mRNA expression for *miR-374b-5p*, *Vegfc*, *Mitf*, *Tyr*, and *S-100* in a mouse melanoma model. (**C**) Western blotting analysis of protein expression for VEGFC, TYR, ERK, pERK, S-100, and MITF proteins in a mouse melanoma model. (**D**) Quantification of protein expression levels (VEGFC, ERK, pERK, pERK/ERK, MITF, TYR, S-100) from Western blot analysis in (**C**), where * denotes *p* < 0.05, ** denotes *p* < 0.01, *** denotes *p* < 0.001, and **** denotes *p* < 0.0001 using one-way ANOVA followed by Tukey’s multiple comparison test.

Based on our in vitro findings that *miR-374b-5p* suppresses VEGFC expression and reduces MAPK pathway activity, we investigated whether similar effects occur in vivo using a murine melanoma model (Figure 3A).

qRT-PCR analysis showed that the expression of *miR-374b-5p* was significantly higher in the *agomiR-374b-5p* group than the control group (Figure 3B, *p* < 0.0001), whereas *miR-374b-5p* expression was significantly reduced in the *antagomiR-374b-5p* group (Figure 3B, *p* < 0.05). These results confirmed the successful manipulation of *miR-374b-5p* expression in vivo.

We then quantified the mRNA expression of *Vegfc* and its downstream genes in tumor tissues. In the *agomiR-374b-5p* group, *Vegfc* mRNA showed a downward trend compared with the control group (Figure 3B, *p* > 0.05). However, *Vegfc* expression was significantly lower in the *agomiR-374b-5p* group than that in the *antagomiR-374b-5p* group (Figure 3B, *p* < 0.001). Conversely, the *antagomiR-374b-5p* group exhibited significantly higher *Vegfc* expression compared with the control group (Figure 3B, *p* < 0.01), indicating that *miR-374b-5p* affects *Vegfc* mRNA abundance in vivo.

For downstream MAPK-related effectors, the *agomiR-374b-5p* group had significantly reduced *Mitf* (*p* < 0.05) and *Tyr* (*p* < 0.05) mRNA levels relative to the control group (Figure 3B), consistent with their reported roles as components of the MAPK signaling network in melanoma. In contrast, *S-100* mRNA showed a significant decrease compared to that in the control group (Figure 3B, *p <* 0.05), consistent with the broader downregulation of MAPK activity and its downstream effectors observed in this study. As expected, *Mitf* (*p* < 0.0001) and *Tyr* (*p* < 0.01) mRNA levels were significantly higher in the *antagomiR-374b-5p* group than in the *agomiR-374b-5p* group (Figure 3B). Together, these data indicate that *miR-374b-5p* modulates downstream gene expression in vivo, an effect that correlates with suppression of *Vegfc.*

To further verify this regulatory mechanism at the protein level, we performed Western blotting analyses on VEGFC, ERK, pERK, MITF, TYR, and S-100 proteins. VEGFC protein expression was significantly lower in the *agomiR-374b-5p* group than in the control group (Figure 3C,D, *p* < 0.05), and significantly higher in the *antagomiR-374b-5p* group compared with the control group (Figure 3C,D, *p* < 0.05). The difference between the *agomiR-374b-5p* and *antagomiR-374b-5p* groups was highly significant (Figure 3C,D, *p* < 0.001). Although *Vegfc* mRNA in vivo did not show strong downregulation, VEGFC protein abundance was clearly reduced by *miR-374b-5p*, consistent with microRNA-mediated translational repression. This divergence highlights the complexity of gene regulation in the tumor microenvironment. Compared with the other groups, there was no significant difference in total ERK protein levels among the groups (Figure 3C,D, *p* > 0.05). The expression of pERK protein in the *agomiR-374b-5p* group was significantly lower than that in the control group (Figure 3C,D, *p* < 0.0001). Furthermore, the pERK/ERK ratio was significantly lower than in the control group (Figure 3C,D, *p* < 0.001), indicating that reduced activation of the MAPK signaling pathway occurs following *miR-374b-5p* overexpression. The downstream of the MITF protein was significantly decreased in the *agomiR-374b-5p* group (Figure 3C,D, *p* < 0.05), as was TYR (Figure 3C,D, *p* < 0.01) compared with the control group. These reductions align with the observed decreases in mRNA and are consistent with changes in MAPK network activity associated with VEGFC modulation. S-100 protein levels in the *agomiR-374b-5p* group were significantly lower than in the control (*p* < 0.05) and *antagomiR-374b-5p* group (*p* < 0.05) (Figure 3C,D), consistent with the observed reduction in other melanoma-associated markers.

Collectively, these in vivo results indicate that *miR-374b-5p* downregulates VEGFC protein and modulates MAPK signaling, accompanied by decreased MITF, TYR, and S-100 expression, consistent with reduced melanoma malignancy.

### 2.6. miR-374b-5p Influences Macrophage Behavior and Polarization-Associated Markers in the Melanoma Microenvironment

**Figure 4:**
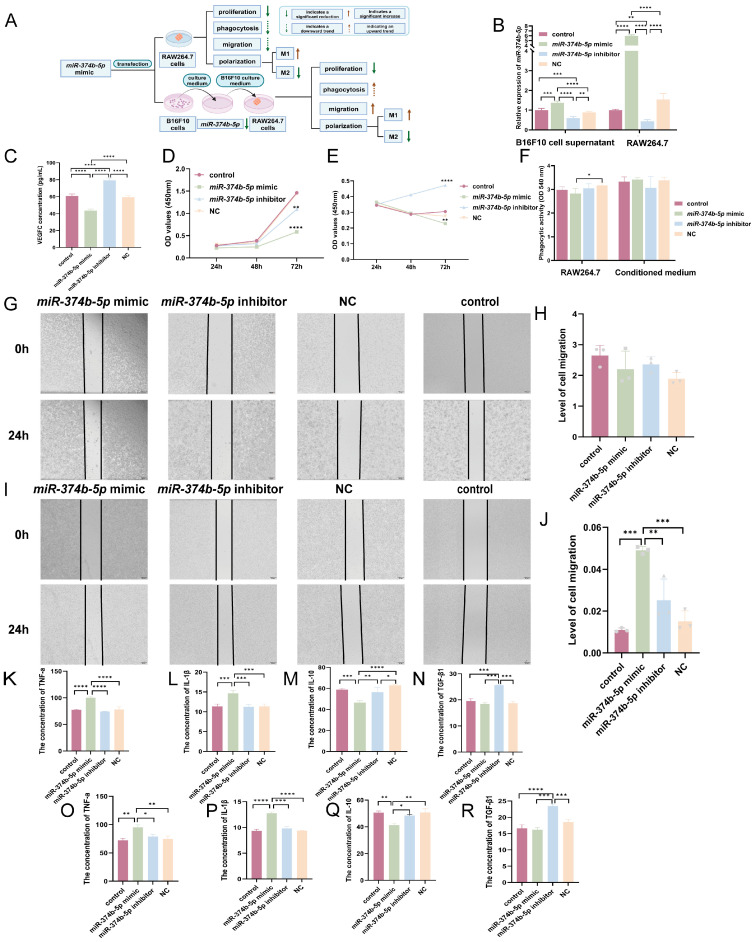
Effect of *miR-374b-5p* on macrophage function in the melanoma microenvironment. (**A**) Schematic diagram of *miR-374b-5p*’s effects on macrophage function in the melanoma microenvironment. (**B**) The levels of *miR-374b-5p* detected in the conditioned medium of transfected B16F10 cells, as well as its intracellular expression levels after transfection into RAW264.7 cells, were determined by qRT-PCR. (**C**) Measurement of VEGFC protein levels in the extracellular medium of B16F10 cells following *miR-374b-5p* transfection, as determined by ELISA. (**D**) CCK-8 assay for determining *miR-374b-5p*-mediated proliferation in transfected macrophages. (**E**) The CCK-8 assay was used to assess the proliferation of conditioned medium cultured macrophages following *miR-374b-5p* transfection in B16F10 cells. (**F**) The Neutral Red Uptake assay was used to assess the phagocytic activity of RAW264.7 cells transfected with *miR-374b-5p*, as well as that of macrophages treated with conditioned medium from B16F10 cells transfected with *miR-374b-5p*. (**G**) Scratch assay to measure the migration of macrophages transfected with *miR-374b-5p*. Scale bar: 200 μm. (**H**) Scratch assay analysis of migratory capacity of macrophages transfected with *miR-374b-5p*. The gray symbols represent individual data values for each group. (**I**) The migration of conditioned medium cultured macrophages was evaluated following *miR-374b-5p* transfection in B16F10 cells through wound healing assays. Scale bar: 200 μm. (**J**) Statistical analysis of wound healing experiments was conducted to evaluate the migration function of conditioned medium-cultured macrophages following *miR-374b-5p* transfection in B16F10 cells. The gray symbols represent individual data values for each group. (**K**–**N**) represent the expression levels of TNF-α, IL-1β, IL-10, and TGF-β1 detected in the culture medium after transfection of *miR-374b-5p* into RAW264.7 cells. (**O**–**R**) represent the expression levels of TNF-α, IL-1β, IL-10, and TGF-β1 in macrophages cultured under conditioned medium after transfection of B16F10 cells with *miR-374b-5p.* Statistical significance was determined by one-way ANOVA with Tukey’s multiple comparison test. * *p* < 0.05, ** *p* < 0.01, *** *p* < 0.001, **** *p* < 0.0001.

As one of the most abundant immune cells in the tumor microenvironment, macrophages exert bidirectional effects on melanoma cells: they can either inhibit tumor progression through immune killing or promote tumor invasion and metastasis. Therefore, we investigated whether *miR-374b-5p* may influence macrophage function upon intracellular delivery and whether melanoma-derived factors regulated by *miR-374b-5p* could affect macrophage responses (Figure 4A).

To investigate whether *miR-374b-5p* can directly modulate macrophage function upon intracellular delivery, we first validated transfection efficiency in RAW264.7 macrophages. qRT-PCR analysis of cell culture supernatants showed a significant increase in extracellular *miR-374b-5p* levels in the mimic group (Figure 4B, *p* < 0.001) and a marked decrease in the inhibitor group compared with the control group (Figure 4B, *p* < 0.001). We further examined whether transfection with this miRNA also altered the extracellular levels of VEGFC protein in B16F10 cell culture supernatants. Consistent with the miRNA data, VEGFC protein levels in the conditioned medium were significantly higher in the inhibitor group (Figure 4C, *p* < 0.0001) and lower in the *miR-374b-5p* mimic group (Figure 4C, *p* < 0.0001) relative to the control group. These findings indicate that *miR-374b-5p* and VEGFC are concurrently present at altered levels in the extracellular environment following *miR-374b-5p* modulation in melanoma cells, revealing a potential mechanism for tumor cell communication with immune cells within the TME.

To assess whether *miR-374b-5p* possesses the intrinsic capacity to directly modulate macrophage function upon intracellular delivery, we first validated transfection efficiency in RAW264.7 macrophages. As expected, macrophages transfected with the mimic exhibited significantly higher *miR-374b-5p* levels than the other groups (Figure 4B, *p* < 0.0001), whereas the inhibitor group showed significantly reduced expression compared with the control group (Figure 4B, *p* < 0.01). These results support the reliability of downstream functional assays.

First, we examined the effect of *miR-374b-5p* on macrophage proliferation. After 72 h of transfection with *miR-374b-5p* mimic, RAW264.7 cells displayed markedly reduced OD values relative to control (Figure 4D, *p* < 0.0001), indicating that *miR-374b-5p* significantly suppressed macrophage proliferation.

To evaluate paracrine effects, we treated RAW264.7 cells with conditioned media from melanoma cells transfected with *miR-374b-5p* mimic or inhibitor. Over a 72 h period, proliferation of RAW264.7 cells exposed to conditioned media from the inhibitor group increased continuously and was significantly higher than that of cells treated with control-conditioned media (Figure 4E, *p* < 0.0001). In contrast, proliferation in the mimic group decreased continuously relative to the control group (Figure 4E, *p* < 0.01). These findings indicate that conditioned media from melanoma cells with altered *miR-374b-5p* expression differentially affect macrophage proliferation.

Second, we examined the effect on macrophage phagocytosis. Direct transfection of macrophages with *miR-374b-5p* produced only a slight, non-significant reduction in phagocytic capacity (Figure 4F, *p* > 0.05). Under B16F10 cell conditioned medium, the mimic group exhibited a small upward trend, but without statistical significance (Figure 4F, *p* > 0.05). These findings suggest that *miR-374b-5p* modulation does not substantially affect macrophage phagocytosis under the conditions tested. Notably, despite reduced proliferation, the phagocytic capacity of remaining macrophages is maintained, ensuring these cells can still perform core innate immune functions within the TME.

Third, we assessed the effect on macrophage migration. When *miR-374b-5p* was introduced directly into macrophages, migration showed only a weak downward trend (Figure 4G,H, *p* > 0.05). However, after 48 h of exposure to conditioned media from *miR-374b-5p*-overexpressing melanoma cells, macrophage migration increased significantly (Figure 4I,J, *p* < 0.001). These results suggest that *miR-374b-5p* may influence macrophage migration indirectly through melanoma cell-derived factors.

Finally, we examined the effect on macrophage polarization. Given the effects of *miR-374b-5p* on macrophage proliferation and migration, we next examined macrophage polarization. Direct transfection of macrophages with the *miR-374b-5p* mimic significantly increased the M1-associated cytokines, TNF-α (*p* < 0.0001) and IL-1β (*p* < 0.001) (Figure 4K,L), while decreasing the M2 cytokine IL-10 (Figure 4M, *p* < 0.001). TGF-β1 showed a mild, non-significant decrease (Figure 4N, *p* > 0.05) compared with the control group. These results indicate that *miR-374b-5p* expression is associated with changes in polarization-related markers toward a more pro-inflammatory profile.

To further assess whether *miR-374b-5p*-overexpressing melanoma cells can influence macrophage phenotype through paracrine signaling, we cultured RAW264.7 cells with conditioned medium from transfected B16F10 cells. The results showed that in the mimic group, TNF-α (*p* < 0.01) and IL-1β (*p* < 0.0001) levels remained significantly higher than those in the control group (Figure 4O,P), IL-10 levels were significantly reduced (Figure 4Q, *p* < 0.01), and TGF-β1 showed a slight but non-significant decrease (Figure 4R, *p* > 0.05). In contrast, the inhibitor group elicited the opposite pattern, including a significant rise in TGF-β1 compared with the control group (Figure 4R, *p* < 0.0001).

**Figure 5:**
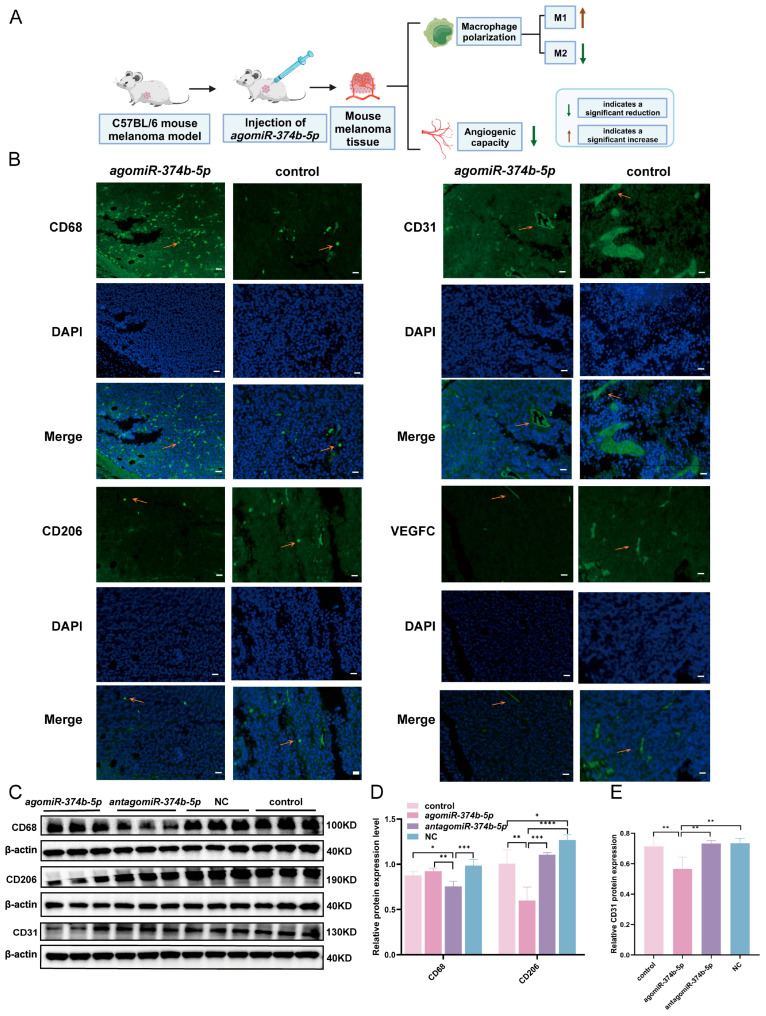
Effects of *miR-374b-5p* on Macrophage Function, Angiogenesis in a Mouse Melanoma Model. (**A**) Schematic diagram of *miR-374b-5p* effects on macrophage function and angiogenesis in a mouse melanoma model. (**B**) Immunofluorescence assay for CD68, CD206, CD31, and VEGFC proteins. The orange arrows indicate the localization of immunofluorescence-labeled proteins. Scale bar: 100 μm. (**C**) Western blotting analysis of protein expression for CD68, CD206, and CD31. (**D**) Quantification of CD68 and CD206 protein expression levels from Western blot analysis in (**C**). (**E**) Quantification of CD31 protein expression levels from Western blot analysis in (**C**), where * denotes *p* < 0.05, ** denotes *p* < 0.01, *** denotes *p* < 0.001, and **** denotes *p* < 0.0001 by one-way ANOVA Tukey’s multiple comparisons.

To determine whether these in vitro observations translate to the tumor context, macrophage infiltration and polarization in tumor tissues were assessed by immunofluorescence and Western blotting (Figure 5A). CD68 (a total macrophage marker) expression showed a mild, non-significant increase in the *agomiR-374b-5p* group (Figure 5B–D, *p* > 0.05), and a significant reduction in the *antagomiR-374b-5p* group compared with the control group (Figure 5B,D, Supplementary Figure S1C, *p* < 0.05). These findings suggest that *miR-374b-5p* has only a modest effect on total macrophage infiltration.

However, CD206 (an M2 macrophage marker) was significantly decreased in the *agomiR-374b-5p* group compared to the control group (Figure 5B–D, *p* < 0.01), consistent with a reduced abundance of M2-like macrophages and a shift toward a more pro-inflammatory profile. This in vivo evidence reveals that *miR-374b-5p* treatment leads to a significant decrease in CD206 expression, consistent with a reduced abundance of M2-like macrophages.

In summary, these findings suggest that *miR-374b-5p* may influence macrophage behavior through both direct and melanoma-mediated mechanisms. The data reveal associations between *miR-374b-5p* expression and shifts in the balance of polarization-associated markers toward a more pro-inflammatory profile, along with effects on macrophage proliferation and migration, while phagocytic function remains largely intact. Collectively, these observations suggest that *miR-374b-5p* may contribute to creating a more favorable immune context within the TME, suggesting potential relevance for understanding immune regulation in melanoma.

### 2.7. miR-374b-5p Reduces Tumor Angiogenesis and VEGFC Expression in Melanoma

To assess the impact of *miR-374b-5p* on angiogenesis and VEGFC expression, we performed immunofluorescence staining and Western blotting on tumor sections (Figure 5A).

Angiogenesis is a critical process in tumor growth and metastasis, providing nutrients and oxygen to tumors and enabling tumor cell dissemination. Quantitative immunofluorescence and Western blotting analyses showed that CD31 (a specific vascular endothelial marker) was significantly reduced in tumors from the *agomiR-374b-5p* group compared with the control group (Figure 5B,C,E, *p* < 0.01), the NC group (Figure 5C,E, Supplementary Figure S2, *p* < 0.01), and the *antagomiR-374b-5p* group (Figure 5C,E, Supplementary Figure S2, *p* < 0.01). These results indicate that *miR-374b-5p* suppresses intratumoral angiogenesis, consistent with its inhibition of the VEGFC-MAPK axis. Reduced vascularization may limit nutrient delivery and restrict vascular conduits for tumor dissemination, potentially contributing to the observed suppression of tumor growth.

Immunofluorescence staining revealed a marked reduction in VEGFC protein levels within the tumor microenvironment of the *agomiR-374b-5p* group compared with the control group (Figure 5B, Supplementary Figure S2). This finding was further corroborated by Western blotting, which confirmed that VEGFC protein expression was significantly decreased following *agomiR-374b-5p* treatment (Figure 3C,D and Figure 5B, *p* < 0.05). Given the well-established role of VEGFC in lymphangiogenesis, these results identify that *miR-374b-5p* downregulates this key pro-lymphangiogenic factor in the tumor microenvironment. Whether this reduction translates into decreased lymphatic vessel density and impaired lymphatic metastasis requires direct assessment using lymphatic endothelial-specific markers such as LYVE-1 or podoplanin in future studies.

## 3. Discussion

In this study, we identify *miR-374b-5p* as a previously uncharacterized regulator associated with melanoma progression and show that its overexpression suppresses tumor growth, accompanied by changes in tumor-intrinsic signaling and features of the tumor microenvironment. Our findings reveal that *miR-374b-5p* directly targets *Vegfc*, which is associated with attenuated MAPK activation, reduced pERK/TYR signaling, and repressed melanoma cell proliferation, migration, and invasion. Beyond these tumor-intrinsic effects, we show that melanoma-derived *miR-374b-5p* is associated with changes in macrophage polarization-related markers within the TME, including reduced M2-like phenotypes, which may contribute to a more pro-inflammatory tumor microenvironment. Collectively, these data suggest that *miR-374b-5p* may restrict melanoma malignancy through coordinated regulation of tumor-intrinsic signaling and features of the tumor microenvironment, with VEGFC acting as an important downstream component.

To investigate the functional consequences of *miR-374b-5p* targeting *Vegfc*, we assessed downstream MAPK activity by measuring pERK levels. Since pERK is the core downstream effector of the MAPK pathway and its expression directly reflects the pathway [33], we assessed pERK levels to evaluate MAPK activity. We found that overexpression of *miR-374b-5p* reduced VEGFC expression, which was accompanied by a decrease in pERK levels. Conversely, inhibition of *miR-374b-5p* expression increased VEGFC levels, and produced a corresponding elevation in pERK. These findings demonstrate a consistent association between *miR-374b-5p*-mediated VEGFC modulation and changes in MAPK pathway activity. Consistent with the established role of pERK in regulating MITF expression [34] and the direct transcriptional activation of TYR by MITF [35], the observed reduction in MITF and TYR protein levels in vivo aligns with the decreased MAPK signaling following *miR-374b-5p*-mediated VEGFC suppression. Notably, this effect was context-dependent, as MITF protein showed limited changes under in vitro culture conditions, highlighting the importance of the tumor microenvironment in facilitating this regulatory axis. These findings demonstrate a consistent association between *miR-374b-5p*-mediated VEGFC downregulation and reduced ERK phosphorylation in B16F10 cells following agomiR treatment. We acknowledge, however, that the effects observed in the *antagomiR-374b-5p* group were more modest. While the directional trends in pERK, MITF, and downstream targets were consistent with the proposed regulatory axis, the changes did not reach statistical significance in all cases. This asymmetry is not unexpected in miRNA loss-of-function studies, where inhibition of an endogenously low-abundance miRNA often produces milder phenotypes compared to overexpression [36]. Nonetheless, the presence of consistent directional trends across multiple downstream markers supports the existence of a regulatory relationship, even if the magnitude of effect is buffered by compensatory mechanisms in vivo. This correlated reduction in MAPK signaling is accompanied by decreased melanoma cell proliferation, migration, and invasion, suggesting that VEGFC may represent an important downstream component of this regulatory axis.

The *miR-374b-5p*/VEGFC axis not only directly regulates tumor cells but also influences the TME, especially through macrophage function. The TME is a critical determinant of melanoma progression. Macrophages demonstrate functional plasticity: M2-polarized macrophages promote immune suppression, tumor development, and metastasis [37], whereas M1-polarized macrophages enhance antitumor immunity [38]. Our comparison of “macrophage-only transfection” versus “Melanoma-conditioned medium on macrophages” showed that direct transfection of *miR-374b-5p* into macrophages reduced proliferation and altered polarization-associated cytokine expression but did not affect migration or phagocytosis. In contrast, macrophages cultured with conditioned medium from *miR-374b-5p-*transfected melanoma cells showed significantly enhanced migration. This effect is likely mediated by soluble factors present in the conditioned medium from *miR-374b-5p*-transfected melanoma cells. The exact nature of these factors, which could include *miR-374b-5p* or its downstream effectors, and the mechanism of their secretion, remain to be fully defined. Notably, the concept that miRNAs can mediate intercellular communication by modulating macrophage function through paracrine mechanisms is well established in the literature. For instance, *miR-374b-5p* secreted by renal tubular epithelial cells enhances M1 polarization by increasing TNF-α and CD86 [39], while *miR-374a-5p* in intracranial aneurysms induces M1 polarization by driving smooth muscle cells to secrete PDGF-BB, IL-1β, IL-6, TNF-α and other macrophage-priming soluble factors [40]. These previous reports provide a conceptual framework supporting the plausibility of our observations, although the specific molecular mediators in our system require further identification. Future studies utilizing tumor-derived exosomes, co-culture systems, or inhibitors of specific secretion pathways will be necessary to more faithfully recapitulate the physiological mode of intercellular miRNA transfer and to definitively characterize the responsible factors. In vivo results further supported this concept: tumor tissues from the *miR-374b-5p* overexpression group showed increased expression of CD68 and decreased expression of CD206. These findings indicate that *miR-374b-5p* modulates the repertoire of soluble factors present in the microenvironment of *miR-374b-5*p-expressing melanoma cells, which is associated with changes in macrophage phenotype within the TME, including increased macrophage accumulation and a shift toward a more pro-inflammatory profile, potentially contributing to enhanced local antitumor immunity. We note that these conclusions are drawn from a limited set of polarization markers; further studies employing additional canonical markers, functional assays, and single-cell approaches will be necessary to fully characterize the nature and extent of macrophage phenotypic changes. Furthermore, the *miR-374b-5p*/VEGFC axis is associated with reduced angiogenesis, which is essential for tumor dissemination and nutrient supply [41]. In vivo analyses (immunofluorescence and Western blotting) confirmed reduced expression of CD31 in the *miR-374b-5p* overexpression group, reflecting decreased intratumor blood vessel density. In addition, VEGFC protein levels were significantly reduced, suggesting a potential impairment of VEGFC-driven pro-lymphangiogenic signaling. In the tumor microenvironment, the level of CD31 expression directly reflects microvessel density. For example, in esophageal squamous cell carcinoma, activation of the Notch signaling pathway can upregulate VEGF expression, inducing endothelial cells to highly express CD31, thereby significantly promoting an increase in tumor microvessel density [42]. VEGFC promotes angiogenesis and lymphangiogenesis in cancers, which has been shown to fuel malignant progression in breast cancer [43], cervical cancer [44], and esophageal cancer [45]. The findings of this study are consistent with these reports. By targeting *Vegfc*, *miR-374b-5p* may contribute to reduced tumor vascularization and thereby potentially limit processes associated with tumor dissemination and complementing its direct antitumor effects. Beyond its effects on macrophage polarization and angiogenesis, the *miR-374b-5p*-mediated modulation of VEGFC and downstream signaling may also intersect with immune checkpoint dynamics. M2-polarized macrophages are known to foster an immunosuppressive microenvironment conducive to T-cell exhaustion and PD-L1 upregulation [46,47], raising the possibility that *miR-374b-5p*-associated changes in macrophage phenotype could influence checkpoint-mediated immune regulation.

Given that our study reveals a consistent association between VEGFC expression and MAPK pathway activity in melanoma, it is plausible that hormone-driven non-genomic MAPK signaling-increasingly recognized as an independent modulator of migration and immune evasion [48]-may converge with or potentiate the VEGFC-related signaling observed here. In this context, the use of male mice in our in vivo experiments is particularly relevant, given emerging evidence that androgens can directly enhance melanoma aggressiveness through non-genomic pathways that intersect with MAPK signaling [49]. For instance, androgen-mediated signaling has been shown to promote tumor cell migration and modulate immune cell function within the tumor microenvironment, potentially exacerbating the malignant phenotypes driven by the VEGFC axis [50]. Thus, our findings in male mice may not only reflect the intrinsic tumor-suppressive effects of *miR-374b-5p* but also hint at its potential to counteract androgen-fueled pro-tumorigenic signals, a hypothesis that warrants direct examination in future studies incorporating androgen manipulation or comparative analyses across sexes. Such interactions could contribute to the well-documented sex differences in melanoma incidence and outcome [51], and warrant future investigations integrating hormonal status with *miR-374b-5p*/VEGFC regulatory dynamics to uncover additional layers of complexity in melanoma progression.

Our data suggest three interconnected mechanisms through which *miR-374b-5p* may influence melanoma progression: (1) Tumor-intrinsic regulation: *miR-374b-5p* targets *Vegfc* and is associated with reduced MAPK signaling, correlating with decreased melanoma cell proliferation, migration, and invasion; (2) TME reprogramming: It is associated with changes in melanoma-derived soluble factors that influence macrophage migration and polarization-related markers; and (3) Association with reduced tumor angiogenesis: *miR-374b-5p* overexpression is associated with decreased CD31 expression and reduced VEGFC levels in tumors.

Despite these strengths, several limitations should be acknowledged. First, while the *agomiR-374b-5p* overexpression experiments produced clear and consistent results, the effects in the antagomiR loss-of-function group were more modest, with some downstream markers showing directional trends that did not reach statistical significance. This asymmetry likely reflects the relatively low basal expression of *miR-374b-5p* in the tumor microenvironment and the intrinsic buffering capacity of signaling pathways, but it also underscores the need for caution when interpreting loss-of-function data in complex in vivo systems. Second, the mechanistic interaction was not confirmed through rescue experiments restoring VEGFC expression or blocking cell receptors. Future studies including rescue experiments and VEGFR-specific inhibition will be necessary to clarify the pathway dependency of this mechanism. Third, conclusions regarding lymphangiogenesis are limited by the use of VEGFC as a readout; VEGFC is a ligand rather than a structural marker, and direct assessment using lymphatic endothelial-specific markers such as LYVE-1 or podoplanin will be necessary to confirm effects on lymphatic vessel density. Furthermore, the observed inhibitory effects of *miR-374b-5p* on the VEGFC/MAPK axis and tumor growth were specific to the male mouse model; future studies including female mice are needed to explore potential sex-dependent interactions. Additionally, although *miR-374b-5p* (via VEGFC regulation) is implicated, the other specific melanoma-derived soluble factors responsible for macrophage modulation remain unidentified. Furthermore, validating this regulatory axis in human melanoma cell lines and clinical samples will be an essential step in future research. Addressing these questions will be the focus of future work.

Collectively, our findings identify a novel role for *miR-374b-5p* as a regulator of melanoma progression that acts through coordinated effects on tumor cells and the tumor microenvironment, with VEGFC serving as a key downstream effector. This study not only elucidates the multi-layered antitumor functions of *miR-374b-5p* in melanoma but also provides a conceptual framework for exploring *miR-374b-5p*-based strategies in melanoma therapy, such as combining *miR-374b-5p*-based approaches with anti-angiogenic agents or immune checkpoint inhibitors, highlighting the potential relevance of *miR-374b-5p* as a target for future melanoma therapeutic strategies.

## 4. Materials and Methods

### 4.1. Ethics Statement

All animal research followed protocols approved by the Animal Experimentation Ethics Committee of Shanxi Agricultural University (Approval No. SXAU-EAW-2024M.PT.001013364). A total of twenty male C57BL/6 specific pathogen-free (SPF) mice, aged six weeks and weighing approximately 18 g, were sourced from SPF (Beijing, China) Biotechnology Co., Ltd. (Beijing, China). At the conclusion of the study, euthanasia was conducted via carbon dioxide asphyxiation, with death confirmed by subsequent cervical dislocation.

### 4.2. Molecular Experimental Methods

#### 4.2.1. Target Prediction

TargetScan (Version 8.0, Whitehead Institute, Cambridge, MA, USA; http://www.targetscan.org/vert_80/, accessed on 5 December 2023) and miRDB (Version 6.0, Washington University School of Medicine, St. Louis, MO, USA; http://mirdb.org/, accessed on 5 December 2023) bioinformatics tools were utilized to search for *miR-374b-5p* target genes. To identify high-confidence candidates for additional validation, the overlap of *miR-374b-5p* targets found in all databases was extracted to select high-confidence candidates for further validation. Binding site maps for potential target genes were generated using the TargetScan technique.

#### 4.2.2. Quantitative Reverse Transcription Polymerase Chain Reaction (qRT-PCR)

Total RNA was isolated from tissue samples using TRIzol reagent (HYCEZMBIO-RHYC01; Wuhan, China). The concentration and purity of the extracted RNA were evaluated using a Nanodrop-2000 spectrophotometer (Thermo Fisher Scientific, Waltham, MA, USA).

As directed by the provider, *miR-374b-5p* was reverse transcribed for miRNA analysis using the miRNA All-In-One cDNA Synthesis Kit (Applied Biological Materials, Zhenjiang, Jiangsu, China). All-In-One 5× RT MasterMix (Applied Biological Materials, Jiangsu, China) was used to reverse transcribe the mRNAs of *Vegfc*, *Mitf*, *Tyr*, and *S-100*. Next, a SYBR Green-based system (Takara, Dalian, China) was used to perform qRT-PCR. The internal reference gene for mRNA targets was *β-actin*, while the endogenous control for *miR-374b-5p* normalization was *U6* snRNA. Supplementary Table S1 contains the primer sequences for *miR-374b-5p*, *U6*, *Vegfc*, *Mitf*, *Tyr*, *S-100*, and *β-actin*.

The 20 µL qRT-PCR reaction included 10 µL of BlasTaq 2× qPCR MM, 0.5 µL of forward and reverse primers (10 µM), 1 µL of fivefold-diluted cDNA template, and 8 µL of nuclease-free water. Amplification was performed in a Stratagene Mx3005P instrument (Agilent Technologies, Santa Clara, CA, USA) using the following cycling conditions: initial denaturation at 95 °C for 3 min, followed by 40 cycles of 95 °C for 15 s and 60 °C for 1 min. Samples were conducted in triplicate, and relative expression levels were evaluated using the 2^−ΔΔCt^ technique.

#### 4.2.3. Dual-Luciferase Reporter Assay

The 3′ untranslated region (3′UTR) of *Vegfc* was amplified by PCR using specific primer sets. The PCR product was purified and subjected to restriction enzyme digestion in a 50 μL reaction system containing 5 μL of 10× buffer, 5 μL of PmirGLO vector, 1 μL of NheI, 1 μL of XbaI, and 38 μL of ddH_2_O. The digestion reaction was incubated at 37 °C for 2 h. After digestion, the vector and insert were ligated, and the positive clones were verified by sequencing.

To perform the reporter assay, 293T cells were plated in 96-well plates and co-transfected with either wild-type or mutant *Vegfc* 3′UTR luciferase reporter constructs, as well as *miR-374b-5p* mimics or negative control RNAs, using GP-transfect-Mate transfection reagent (GenePharma, Shanghai, China). After 36 h, firefly and Renilla luciferase activity were measured sequentially using a Dual-Luciferase Reporter Assay Kit (Beyotime, Shanghai, China), following the manufacturer’s instructions. Relative luciferase activity was calculated as the ratio of firefly to Renilla luminescence. Supplementary Figure S1A shows the sequence of the modified *Vegfc* 3′UTR region.

#### 4.2.4. Western Blotting Analysis for Protein Expression Detection

Total proteins were extracted from B16F10 cells and mouse tumor tissues using a protein commercial extraction kit (Solarbio, Beijing, China). Protein concentrations were quantified using a BCA assay kit (Sangon Biotech, Shanghai, China). Heat-denatured aliquots of 40 µg of protein each lane were resolved by SDS-PAGE and transferred onto PVDF membranes (Millipore, Billerica, MA, USA).

Membranes were blocked with 5% non-fat dried milk for 90 min before being probed overnight at 4 °C with the primary antibodies diluted 1:2000 in TBST: rabbit monoclonal anti-VEGFC (Immunoway-YT5297, Plano, TX, USA), rabbit monoclonal anti-ERK (ERK1/2; Immunoway-YM8336, TX, USA), rabbit monoclonal anti-pERK (ERK1/2 Phospho Thr202/Tyr204; Immunoway-YM8452, TX, USA), mouse monoclonal anti-TYR (Immunoway-YM4942, TX, USA), rabbit monoclonal anti-MITF (Immunoway-YM8062, TX, USA), anti-S-100 (Immunoway-YN5481, TX, USA), anti-CD31 (Immunoway-YM8027, TX, USA), anti-CD68 (Immunoway-YM8367, TX, USA), and anti-CD206 (Immunoway-YM8349, TX, USA). Rabbit monoclonal anti-β-actin (CWBIO-CW0096M; 1:3000 in TBST; Taizhou, China) served as the loading control.

After two 10 min TBST washes, membranes were incubated for 1 h at 25 °C with horseradish peroxidase (HRP)-conjugated goat anti-rabbit or anti-mouse secondary antibodies (CWBIO; 1:7000 in TBST, Taizhou, China). After three more 5 min TBST washes, immunoreactive bands were identified with a SuperKine ECL substrate (AbbKine, Wuhan, China). Images were taken with a ChemiDoc XRS+ imaging equipment (Bio-Rad, Hercules, CA, USA) and band intensities were measured using Image-Pro Plus software (Version 7.0, Media Cybernetics, Inc., Rockville, MD, USA).

### 4.3. Cellular Experimental Methods

#### 4.3.1. Cell Culture

Mouse B16F10 (cat. no. T1254), human 293T (cat. no. LV010), and mouse RAW264.7 (cat. no. T9096) cell lines were sourced from Applied Biological Materials Inc. All cell lines were maintained in Dulbecco’s Modified Eagle Medium (DMEM) supplemented with 10% fetal bovine serum (FBS) and 1% penicillin-streptomycin. Cells were routinely passaged upon reaching 80–90% confluence. Subculturing was performed by aspirating the media and briefly treating the cell monolayer with 1 mL of trypsin in a T25 flask. Following full separation at room temperature, the enzymatic reaction was stopped by adding complete medium. The cell suspension was centrifuged at 1000× *g* for 5 min. After discarding the supernatant, the pellet was resuspended in fresh complete medium and seeded into six-well plates for further incubation at 37 °C under a 5% CO_2_ atmosphere.

#### 4.3.2. Cell Transfection

GenePharma (Shanghai, China) produced the *miR-374b-5p* mimic, *miR-374b-5p* inhibitor, and a scrambled negative control oligonucleotide. The sequences were as follows: *miR-374b-5p* mimic sense: 5′-AUAUAAUACAACCUGCUAAGUG-3′, antisense: 5′-ACUUAGCAGGUUGUAUUAUAUU-3′; *miR-374b-5p* inhibitor: 5′-CACUUAGCAGGUUGUAUUAUA-3′; negative control: 5′-UUCUCCGAACGUGUCACGUTT-3′, antisense: 5′-ACGUGACACGUUCGGAGAATT-3′. Transfections were performed using GP-transfect-Mate reagent (GenePharma, Shanghai, China) as directed. In brief, cells were plated in six-well plates and transfected at 50–60% confluence. Each nucleic acid (*miR-374b-5p* mimic at a final concentration of 75 nM, *miR-374b-5p* inhibitor at 75 nM, and negative control at 75 nM) was mixed with transfection reagent in serum-free DMEM, incubated for 15 min at room temperature to form complexes, and then administered dropwise to cells. After 6 h, the transfection mixture was replaced with fresh complete medium.

#### 4.3.3. Conditioned Medium Assay

B16F10 cells were plated in 6-well plates at 1 × 10^5^ cells per well. Following attachment, transfections were performed using GP-Transfection-Reagent (GenePharma, Shanghai, China) per the manufacturer’s instructions. Cell viability (>90%) and the absence of obvious morphological abnormalities were confirmed 48 h after transfection using trypan blue staining and microscopic observation, respectively. The culture supernatant from these transfected B16F10 cells was then collected. Meanwhile, RAW264.7 cells were seeded in 12-well plates at 3 × 10^5^ cells per well and grown to approximately 80% confluence. The collected conditioned medium from B16F10 cells was applied to the RAW264.7 monolayers, which were then incubated for 48 h at 37 °C in a 5% CO_2_ atmosphere. This approach was employed to assess the effects of conditioned medium from *miR-374b-5p*-overexpressing B16F10 cells on RAW264.7 macrophage phenotypes.

### 4.4. Cell Function Assays

#### 4.4.1. Cell Proliferation Assay (Cell Counting Kit-8, CCK-8)

At 24 h post-transfection, cells were seeded in 96-well plates at a density of 5 × 10^3^ cells/well, with six replicate wells per group. Plates were incubated at 37 °C with 5% CO_2_ for 24, 48, 72, and 96 h. At each time point, the medium was withdrawn and replaced with 90 μL of new complete medium and 10 μL of CCK-8 reagent (Dojindo, Tokyo, Japan) in each well. After one hour of incubation, absorbance at 450 nm was measured with a microplate reader (Thermo Fisher Scientific, Waltham, MA, USA).

#### 4.4.2. Plate Colony Formation Assay

At 24 h post-transfection, cells were plated at a low density of 500 cells per well in a fresh 6-well plate. The plates were maintained in a humidified 37 °C incubator with 5% CO_2_ for one week, with routine microscopic observation every 12 h. Incubation was terminated once more than 50 distinct colonies had formed per well. The culture medium was then aspirated, and the cells were rinsed gently three times with 0.01 M phosphate-buffered saline (PBS, pH 7.4). Colonies were fixed with 1 mL of methanol per well for 30 min, followed by staining with 1 mL of Giemsa solution for 20 min. After three additional PBS washes, the plates were air-dried and imaged using a gel documentation system (Bio-Rad, USA). The colony formation rate was determined as: (Number of colonies/Number of cells seeded) × 100%.

#### 4.4.3. Cell Migration Assay

Twenty-four hours post-transfection, cells were plated in 24-well plates at a density of 5 × 10^5^ cells per well and cultured until reaching full confluence. A uniform linear scratch was then introduced across the cell monolayer using a sterile 1 mL pipette tip. After gently washing three times with PBS to remove dislodged cells, 1 mL of serum-free DMEM was added to each well. The initial wound width was recorded by capturing images at 40× magnification immediately (0 h). Plates were returned to the 37 °C, 5% CO_2_ incubator for 24 h, after which images of the same fields were acquired again. The migratory capacity was quantified by measuring the change in the wound area using ImageJ software (Version 1.8.0, National Institutes of Health, Bethesda, MD, USA) and expressed as the percentage of wound closure: [(Scratch area at 0 h − Scratch area at 24 h)/Scratch area at 0 h] × 100%.

#### 4.4.4. Cell Invasion Assay

Matrigel Matrix Gel (Corning, Shanghai, China) was thawed overnight at 4 °C after removal from −20 °C storage. Cells were serum-starved for 24 h to synchronize growth prior to the assay. The thawed Matrigel was diluted 1:8 with ice-cold serum-free medium, and 60 μL of the diluted gel was evenly applied to the upper surface of each Transwell insert (8 μm pore, Corning, Corning, NY, USA). Inserts were then incubated at 37 °C for 3 h to allow gel polymerization. Following serum starvation, cells were harvested and resuspended in serum-free medium at a density of 8 × 10^4^ cells per 100 μL. A total of 8 × 10^4^ cells in 100 μL suspension were added to the upper chamber of each Matrigel-coated insert. The lower chamber was filled with DMEM containing 20% FBS and 1% penicillin-streptomycin as a chemoattractant. The plate was incubated for 48 h at 37 °C in 5% CO_2_. After incubation, medium was removed and the inserts were washed twice with PBS. Cells on the lower surface of the membrane were fixed with 4% paraformaldehyde (PFA) for 20 min, washed, and stained with 0.1% crystal violet for 25 min. Non-invading cells on the upper side of the membrane were gently removed with a cotton swab. The inserts were rinsed twice with distilled water, air-dried, and imaged under an inverted microscope. Five randomly selected fields per insert were captured, and invading cells were counted using ImageJ software.

#### 4.4.5. Cell Phagocytosis Assay

Twenty-four hours after transfection, cells were plated in 96-well plates at a density of 1 × 10^4^ cells per well and cultured until a confluent monolayer formed. The medium was then aspirated, and each well was gently rinsed with 100 µL of PBS. Subsequently, 200 µL of 0.1% neutral red solution (Solarbio, Beijing, China) was added to each well, and the plates were returned to the 37 °C, 5% CO_2_ incubator for 3 h to allow phagocytic uptake of the dye. Following the incubation period, the neutral red solution was removed, and 200 µL of cell lysis buffer (a 1:1 [*v*/*v*] mixture of glacial acetic acid and absolute ethanol) was added to each well. The plates were kept at room temperature until complete cell lysis was achieved. Absorbance was then measured at 540 nm using a microplate reader (Thermo Fisher Scientific, Waltham, MA, USA).

### 4.5. In Vivo Mouse Experiments

#### 4.5.1. Functional Assay of miR-374b-5p in Mice

B16F10 mouse melanoma cells were harvested after 24 h of culture, washed three times with PBS, and detached using 1 mL of trypsin for 2 min. The digestion was stopped by adding complete medium, and the cell suspension was centrifuged at 1000× *g* for 5 min. The pellet was resuspended in PBS to a final density of 1 × 10^7^ cells/mL. Each mouse received a subcutaneous injection of 100 μL of this suspension into the right forelimb axilla to induce tumor formation.

Tumor models were considered established when the average tumor volume reached approximately 62.5 mm^3^ (around 7 days post-injection). Mice were then randomly allocated into four groups (*n* = 5 per group): Control group (tumor-bearing mice without *miR-374b-5p* treatment), *agomiR-374b-5p* group (tumor-bearing mice treated with *agomiR-374b-5p*), *antagomiR-374b-5p* group (tumor-bearing mice treated with *antagomiR-374b-5p*), and NC group (tumor-bearing mice treated with negative control RNA). Tumor volume was monitored and calculated using the formula V = (a × b^2^)/2, where a represents the longest diameter and b the perpendicular shorter diameter.

Seven days after establishment of the melanoma model, the miRNA mimic and inhibitor were administered by multipoint intratumoral injection. Except for the control group, each treatment group received one intratumoral injection per day for seven consecutive days, with 50 μL RNA solution injected each daily administration. Specifically, the *agomiR-374b-5p*, *antagomiR-374b-5p* and negative control (NC) oligonucleotides were used in accordance with the manufacturer’s instructions (LifeSpan, Shanghai, China). The sequences of *miR-374b-5p* mimic and inhibitor are as follows: *agomiR-374b-5p* sense: 5′-AUAUAAUACAACCUGCUAAGUG-3′, antisense: 5′-CUUAGCAGGUUGUAUUAUAUUU-3′; *antagomiR-374b-5p*: 5′-CACUUAGCAGGUUGUAUUAUAU-3′ (chemically modified with 2′-O-methyl and phosphorothioate linkages for in vivo stability). For each daily injection, mice received RNA solutions containing 1 nmol *agomiR-374b-5p*, 5 nmol *antagomiR-374b-5p*, or 1 nmol NC, respectively.

Twenty-four hours following the final (seventh) daily injection, tumors were measured using Vernier calipers. The relative tumor volume was determined as Vt/V0, where Vt is the volume post-treatment and V0 is the pretreatment volume [52]. Subsequently, mice were euthanized, and the subcutaneous tumors were dissected and promptly frozen at −80 °C for subsequent molecular and histopathological examination.

#### 4.5.2. Immunofluorescence Analysis

Excised tumor tissues were fixed in 4% PFA at 4 °C for 24 h. After fixation, samples were dehydrated through a graded ethanol series, cleared in xylene, and embedded in paraffin. Sections of 6 µm thickness were cut and mounted on charged glass slides (Sigma-Aldrich, St. Louis, MO, USA). Following deparaffinization in xylene and rehydration through decreasing ethanol concentrations, heat-induced epitope retrieval was performed by microwaving sections in Tris-EDTA antigen retrieval solution (pH 9.0; Leaguee, Beijing, China) for 20 min. After cooling and washing with PBS, sections were blocked with 3% bovine serum albumin (BSA) for 2 h at room temperature to minimize nonspecific binding. The blocked sections were then incubated overnight at 4 °C with the following primary antibodies diluted 1:500 in 3% BSA: anti-CD31 (Immunoway, YM8027, TX, USA), anti-CD68 (Immunoway, YM8367, TX, USA), and anti-CD206 (Immunoway, YT5640, TX, USA). After three PBS washes, sections were incubated for 1 h in the dark with a fluorescent secondary antibody (AbFluor 488, Immunoway, TX, USA; 1:500 in 3% BSA) at room temperature. Nuclei were counterstained with DAPI (Sigma-Aldrich) for 10 min. Finally, slides were coverslipped with antifade mounting medium and visualized using a Nikon ECLIPSE Ts2R inverted fluorescence microscope (Nikon Corporation, Tokyo, Japan).

#### 4.5.3. ELISA Assay

Tissue samples were homogenized in ice-cold PBS (pH 7.4) and centrifuged at 1000× *g* for 10 min at 4 °C. The supernatant was collected for analysis using a commercial ELISA kit (Byabscience Biotechnology Co., Ltd., Nanjing, China) according to the manufacturer’s protocol, including kits for VEGFC (BY-EM228284), M1 macrophage markers (TNF-α, BY-EM220852; IL-1β, BY-EM220174), and M2 macrophage markers (IL-10, BY-EM220162; TGF-β1, BY-EM220862). Briefly, standards and samples were added to a 96-well plate in duplicate. Sample wells received 40 µL of diluent and 10 µL of supernatant, while standard wells were filled with 50 µL of serially diluted standards. Blank wells contained diluent only. After adding 100 µL of horseradish peroxidase-conjugated detection antibody to each well (except blanks), the plate was incubated at 37 °C for 1 h. Wells were then washed five times with 400 µL wash buffer. Subsequently, 50 µL each of chromogen substrates A and B were added, followed by a 15 min incubation at 37 °C in the dark. The reaction was stopped with 50 µL stop solution, and absorbance was measured at 450 nm within 15 min using a microplate reader (Thermo Fisher Scientific, Waltham, MA, USA). Target protein concentrations were interpolated from a standard curve.

### 4.6. Statistical Analyses

Data are presented as mean ± standard error (SE). A threshold of *p* < 0.05 was considered statistically significant. For comparisons among multiple groups, one-way analysis of variance (ANOVA) was applied, followed by Tukey’s test for post hoc pairwise comparisons. All analyses and graphical representations were performed using SPSS 26.0 (IBM, Armonk, NY, USA), ImageJ (version 2.14.0/1.54f, National Institutes of Health,, Bethesda, MD, USA), and GraphPad Prism 9.5 (GraphPad Software, San Diego, CA, USA).

## 5. Conclusions

This study identifies *miR-374b-5p* as a key regulator of melanoma progression that suppresses tumor cell proliferation, migration, and invasion in association with VEGFC downregulation and reduced MAPK signaling, while reprogramming the tumor microenvironment toward an antitumor state. It also inhibits tumor angiogenesis and downregulates VEGFC, a master driver of lymphangiogenesis, thereby potentially limiting metastatic potential. Taken together, our findings reveal a multi-layered antitumor mechanism centered on the *miR-374b-5p*/VEGFC axis, underscoring its potential as a therapeutic target for melanoma.

## Data Availability

The original contributions presented in this study are included in the article/Supplementary Materials. Further inquiries can be directed to the corresponding authors.

## Supplementary Materials

The following supporting information can be downloaded at: https://www.mdpi.com/article/10.3390/ijms27062854/s1.

## Abbreviations

The following abbreviations are used in this manuscript:

VEGFC	Vascular Endothelial Growth Factor C
MITF	Microphthalmia-associated Transcription Factor
TYR	Tyrosinase
*miR-374b-5p*	*microRNA-374b-5p*
