# Supplementary material for: miR-374b-5p Modulates Melanoma Progression by Targeting VEGFC and Regulating MAPK Signaling in the Tumor Microenvironment
Source: Int J Mol Sci. 2026 Mar 21;27(6):2854. doi: 10.3390/ijms27062854 (PMC13026663; doi:10.3390/ijms27062854)
Supplement: Supplementary file 1 [file ijms-27-02854-s001.zip › ijms-4120419-supplementary.pdf]

Supplementary

Table S1. Primer sequence of target genes.

Gene	Primer	Length
U6	F：GGAACGATACAGAGAAGATTAGC<br>R：TGGAACGCTTCACGAATTTGCG	68
miR-374b-5p	F：AUAUAAUACAACCUGCUAAGUG	-
β-actin	F：AGGGAAATCGTGCGTGAC<br>R：CATACCCAAGAAGGAAGGCT	192
Vegfc	F：CCAACAAGGAGCTGGATGAA<br>R：GGCTCCACATGAATTAGGGAA	146
Mitf	F：GACTTTCCCTTATCCCATCCAC<br>R：TGGTGCTGTACAAGTTCCTG	109
Tyr	F：TGAAGCACCAGGGTTTCTG<br>R：GCATCTCTCCAATCCCAGTATG	117
S-100	F：CCATGGAGACCCTCATCAAT<br>R：GTAGCAGGTCTTTCAGTTCTTTC	93

Table S2. KEGG pathway enrichment analysis of target genes of *miR-374b-5p.*

ID	Description	Proteins
mmu05200	Pathways in cancer	Wnt3, Pparg, Sp1, Akt1, Rala, Pten, Crk, Fgf18, Hes1, Bmp2, Ccne2, Gnb2, Tgfa, Lrp6, Vegfc, Gadd45a, Lpar1, Foxo1, Wnt5a, Fzd5, Apc, Ralgds, Fgfr2
mmu05224	Breast cancer	Wnt3, Sp1, Akt1, Pten, Fgf18, Hes1, Lrp6, Gadd45a, Wnt5a, Fzd5, Apc
mmu04010	MAPK signaling pathway	Ngfr, Akt1, Crk, Dusp6, Fgf18, Map2k6, Tgfa, Vegfc, Rasa2, Map2k4, Gadd45a, Rasa1, Ntf3, Fgfr2
mmu04014	Ras signaling pathway	Ngfr, Akt1, Rala, Fgf18, Gnb2, Tgfa, Vegfc, Rasa2, Ralgds, Rasa1, Ntf3, Fgfr2
mmu05226	Gastric cancer	Wnt3, Akt1, Fgf18, Ccne2, Lrp6, Gadd45a, Wnt5a, Fzd5, Apc, Fgfr2
mmu04015	Rap1 signaling pathway	Ngfr, Akt1, Rala, Crk, Fgf18, Map2k6, Vegfc, Lpar1, Pard6b, Ralgds, Fgfr2
mmu04550	Signaling pathways regulating pluripotency of stem cells	Wnt3, Akt1, Smarcad1, Kat6a, Wnt5a, Fzd5, Apc, Fgfr2, Acvr2b
mmu05225	Hepatocellular carcinoma	Wnt3, Akt1, Pten, Tgfa, Lrp6, Gadd45a, Wnt5a, Fzd5, Apc, Arid2
mmu05217	Basal cell carcinoma	Wnt3, Bmp2, Gadd45a, Wnt5a, Fzd5, Apc
mmu05202	Transcriptional misregulation in cancer	Ngfr, Pparg, Sp1, Dusp6, Nr4a3, Gadd45a, Foxo1, Aff1, Cebpb, Hoxa10
mmu04350	TGF-beta signaling pathway	Sp1, Bmp2, Smad6, Pitx2, Neo1, Acvr2b, Tfdp1

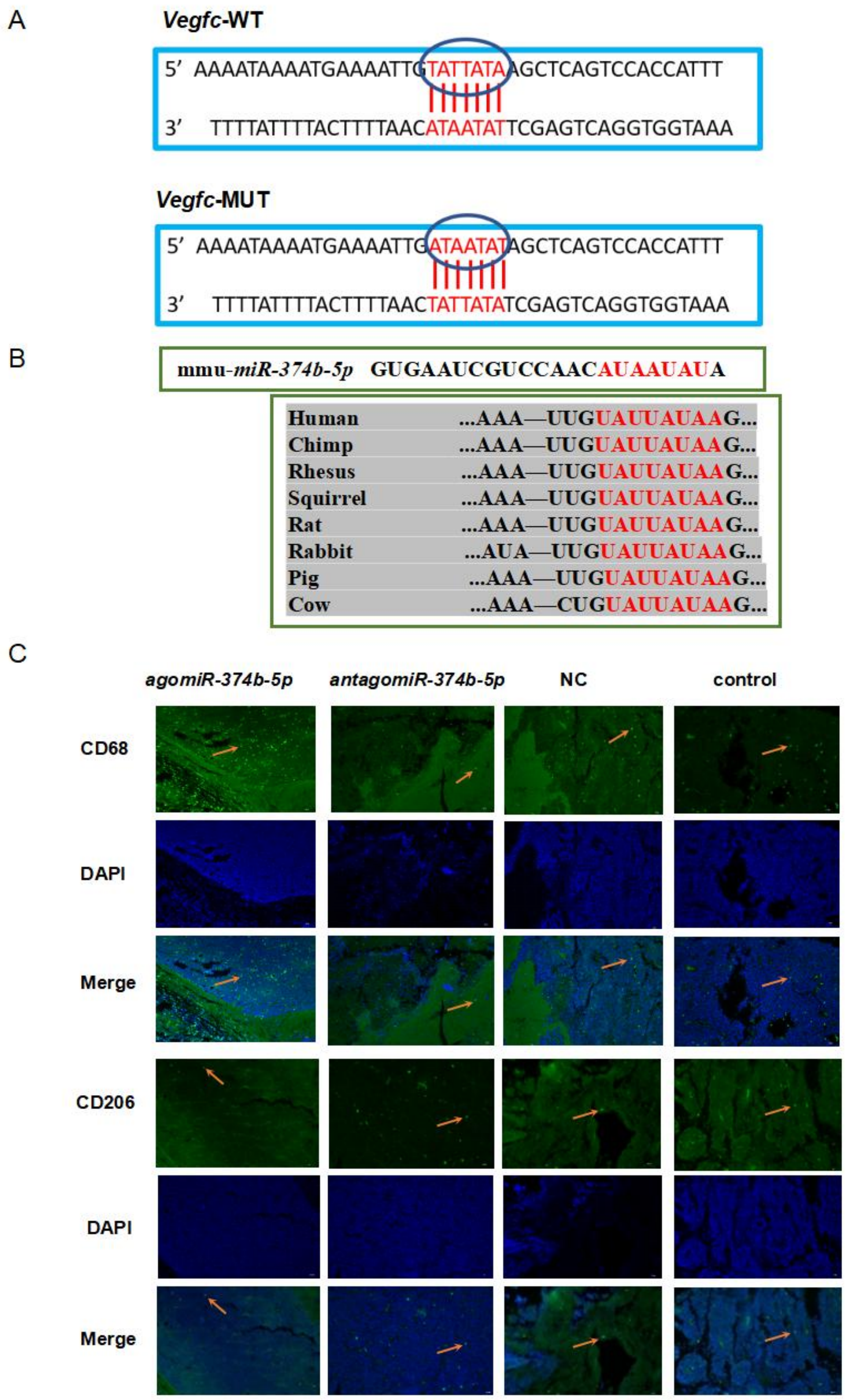

Figure S1. A *Vegfc* vector multiple cloning site insertion sequence information. **B** Conservation of *miR-374b-5p* targeting the *Vegfc* gene across multiple species. **C** Immunofluorescence assay for CD68 and CD206 proteins. Scale bar: 100 μm.

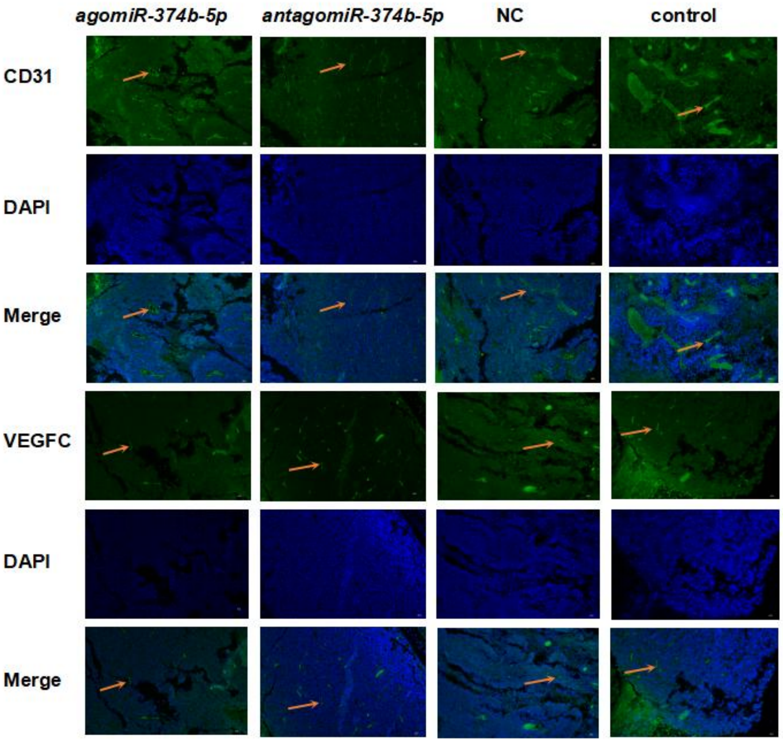

Figure S2. Immunofluorescence assay for CD31 and VEGFC proteins. Scale bar: 100 μm.
